# Impact of COVID-19 on the association between pulse oximetry and arterial oxygenation in patients with acute respiratory distress syndrome

**DOI:** 10.1038/s41598-021-02634-z

**Published:** 2022-01-27

**Authors:** Lee S. Nguyen, Marion Helias, Lisa Raia, Estelle Nicolas, Paul Jaubert, Sarah Benghanem, Zakaria Ait Hamou, Pierre Dupland, Julien Charpentier, Frédéric Pène, Alain Cariou, Jean-Paul Mira, Jean-Daniel Chiche, Mathieu Jozwiak

**Affiliations:** 1 Hôpital Cochin, Service de Médecine Intensive Réanimation, Assistance Publique - Hôpitaux de Paris, Hôpitaux Universitaires Paris-Centre, 27 rue du faubourg Saint Jacques, 75014 Paris, France; 2 Université de Paris, Paris, France; 3 Research and Innovation Department, CMC Ambroise Paré, Neuilly-sur-Seine, France; 4 Equipe 2 CARRES, UR2CA - Unité de Recherche Clinique Côte d’Azur, Université Côte d’Azur UCA, Nice, France

**Keywords:** Diagnosis, Therapeutics

## Abstract

Managing patients with acute respiratory distress syndrome (ARDS) requires frequent changes in mechanical ventilator respiratory settings to optimize arterial oxygenation assessed by arterial oxygen partial pressure (PaO_2_) and saturation (SaO_2_). Pulse oxymetry (SpO_2_) has been suggested as a non-invasive surrogate for arterial oxygenation however its accuracy in COVID-19 patients is unknown. In this study, we aimed to investigate the influence of COVID-19 status on the association between SpO_2_ and arterial oxygenation. We prospectively included patients with ARDS and compared COVID-19 to non-COVID-19 patients, regarding SpO_2_ and concomitant arterial oxygenation (SaO_2_ and PaO_2_) measurements, and their association. Bias was defined as mean difference between SpO_2_ and SaO_2_ measurements. Occult hypoxemia was defined as a SpO_2_ ≥ 92% while concomitant SaO_2_ < 88%. Multiple linear regression models were built to account for confounders. We also assessed concordance between positive end-expiratory pressure (PEEP) trial-induced changes in SpO_2_ and in arterial oxygenation. We included 55 patients, among them 26 (47%) with COVID-19. Overall, SpO_2_ and SaO_2_ measurements were correlated (r = 0.70; *p* < 0.0001), however less so in COVID-19 than in non-COVID-19 patients (r = 0.55, *p* < 0.0001 *vs.* r = 0.84, *p* < 0.0001, *p* = 0.002 for intergroup comparison). Bias was + 1.1%, greater in COVID-19 than in non-COVID-19 patients (2.0 *vs.* 0.3%; *p* = 0.02). In multivariate analysis, bias was associated with COVID-19 status (unstandardized β = 1.77, 95%CI = 0.38–3.15, *p* = 0.01), ethnic group and ARDS severity. Occult hypoxemia occurred in 5.5% of measurements (7.7% in COVID-19 patients *vs.* 3.4% in non-COVID-19 patients, *p* = 0.42). Concordance rate between PEEP trial-induced changes in SpO_2_ and SaO_2_ was 84%, however less so in COVID-19 than in non-COVID-19 patients (69% *vs.* 97%, respectively). Similar results were observed for PaO_2_ regarding correlations, bias, and concordance with SpO_2_ changes. In patients with ARDS, SpO_2_ was associated with arterial oxygenation, but COVID-19 status significantly altered this association.

## Introduction

From December 2019, a worldwide pandemic with an kk coronavirus SARS-CoV-2 is responsible for Coronavirus disease (COVID-19^1^. Up to two-thirds of hospitalized COVID-19 patients develop severe pneumonia leading to an acute respiratory distress syndrome (ARDS)^2,3^. ARDS is characterized by an impairment in arterial oxygenation, leading to profound hypoxemia. Hence, the ratio between arterial oxygen partial pressure (PaO_2_) and inspired fraction in oxygen (FiO_2_), delivered by the mechanical ventilator, is a crucial in ARDS management. Yet, PaO_2_ requires arterial blood gas analyses and cannot be continuously monitored. It may be approximated by arterial oxygen saturation (SaO_2_), which in turn, may be estimated by pulse oximetry (SpO_2_), a continuous non-invasive measurement^4^. The latter showed benefit in decreasing complications related to intraoperative hypoxemia incidents^5^, rate of unplanned intensive care unit (ICU) admissions of surgical patients^6^ and number arterial blood gas analysis^7–10^.

Managing patients with ARDS requires frequent changes in mechanical ventilator respiratory settings. While arterial oxygenation is paramount towards guiding the fine-tuning of mechanical ventilators in ARDS, SpO_2_ has been suggested as surrogate for arterial oxygenation^11–13^. Known caveats of SpO_2_ mostly lie in its accuracy in ICU due to confounding factors : hypoxemia^14–18^, vasoconstriction with microcirculatory disorders^19^, vasopressor treatments^15,20^ and racial bias^21^.

Since some COVID-19 patients are black^22^22/11/2021 13:53:00, severely hypoxemic^23^ and may also experience microcirculatory disorders^24^, the main goal of the following study was to investigate the impact of COVID-19 status on the association between SpO_2_ and arterial oxygenation (SaO_2_ and PaO_2_) in patients with ARDS. Furthermore, we assessed whether SpO_2_ changes could track changes in arterial oxygenation (SaO_2_ and PaO_2_) during positive end-expiratory pressure (PEEP) trial.

## Methods

This prospective and observational study was conducted in a 24-bed ICU of a French university hospital between March 2019 and July 2020 for non COVID-19 patients and in the first pandemic wave (March–April 2020) for COVID-19 patients, in accordance with relevant guidelines and regulations.

### Patients and data collection

We included all consecutive patients admitted in our ICU department for acute respiratory failure and presented ARDS criteria, according to Berlin definition^26^ at the initial phase of ventilatory management (within one hour after intubation). Exclusion criteria were those related to known causes of low-reliability of SpO_2_ measurements: (i) low-quality SpO_2_ signal assessed on the SpO_2_ curve aspect and (ii), patients with nail varnish, methaemoglobinemia or carbon monoxide poisoning^4^ and (iii), patients with marked movements leading to SpO_2_ signal artefacts^27^.

ARDS severity was categorized as mild for PaO_2_/FiO_2_ ratio between 200 and 300 mmHg, moderate between 100 and 200 mmHg, and severe, under 100 mmHg^26^.

Data were prospectively collected using a clinical software allowing data management and extraction, Centricity Critical Care (General Electric Healthcare, Massachusetts, United States of America).

### SpO_2_ and SaO_2_ measurements

SpO_2_ was continuously measured using a finger or ear probe of a last generation pulse oximeter (Masimoset®, Masimo Corporation, Irvine, CA, USA). The location of SpO_2_ probe was left at the discretion of nurses to obtain the best possible SpO_2_ signal. The pulse oximeter was connected to patients’ monitor (GE Healthcare, Chicago, Il, USA) and SpO_2_ signal maximized on the monitor screen. SpO_2_ measurement was recorded at the time the arterial blood gas was performed by the patient’s nurse.

A blood sample was obtained from radial or femoral arterial catheter, not necessarily located on the same side as SpO_2_ probe. SaO_2_ was measured with the ICU blood gas analyser (ABL800, Radiometer®, Copenhagen, Denmark) and thus obtained within five minutes of blood sampling.

Occult hypoxemia was defined as SaO_2_ < 88%, while concomitant SpO_2_ measure was ≥ 92%^21^.

### Ventilatory settings and respiratory measurements

All patients were placed in 45-degree semi-recumbent position and mechanically ventilated (CARESCAPE R860, GE Healthcare, Chicago, Il, USA) in pressure regulated volume control mode. Tidal volume was set at 6 mL/kg of predicted body weight. Respiratory rate and inspiratory/expiratory times ratio were adjusted to prevent hypercapnia (pH goal: 7.30–7.45) and to avoid dynamic hyperinflation, without exceeding a respiratory rate of 35 breaths/min^12,28^. The fraction of inspired oxygen (FiO_2_) was adjusted to obtain a SpO_2_ ≥ 90%^29^. An airway humidification system was used in all patients.

The plateau pressure was calculated during a 5-s inspiratory hold and total PEEP during a 5-s expiratory hold. The driving pressure was calculated as the difference between plateau pressure and total PEEP. The static respiratory system compliance was calculated as tidal volume/(plateau pressure–total PEEP).

### PEEP trial description

Since only patients with moderate to severe ARDS with a high potential for lung recruitment benefit from high PEEP strategies and since the amount of potentially recruitable lung vary widely in the population^30^, we systematically performed a PEEP trial in all patients with ARDS to individualize at best the initial PEEP level^29^. According to local protocols, PEEP level was initially set at 5 cmH_2_O^31^ and a first set of measurements, including SpO_2_, SaO_2_ and PaO_2_ was performed. Then, PEEP was increased to 15 cmH2O and a second set of measurements was performed. All measurements were recorded after a 10-min period of stabilization^32^ and two couples of simultaneous SpO_2_ and SaO_2_ measurements were obtained per patient. Other ventilatory settings were unchanged during the study period.

### COVID-19 diagnosis

All patients were confirmed with SARS-Cov-2 using routine RT-PCR methodology, with two sets: either Allplex® 2019-nCoV assay (Seegene, Seoul, South Korea) with Microlab NIMBUS® extractor (Hamilton Bonaduz AG, Rapperswil-Jona, Switzerland) and CFX96® thermocycler (Bio-Rad laboratories, Hercules, California, United States of America); or RealStar® SARS-CoV-2 RT-PCR Kit 1.0 assay (Altona Diagnostics, Hamburg, Germany) with QIAsymphony SP® extractor (QIAGEN, Hilden, Germany) and QuantStudio® thermocycler (Thermo Fisher Scientific, Waltham, Massachusetts, United States of America). RT-PCR was performed on nasopharyngeal swabs or on distal bronchial samples.

### Statistical analysis

Normality distribution of continuous variables was tested using the Agostino-Pearson test. Continuous variables were expressed as mean (standard deviation) or median [interquartile] and categorical variables as counts (percentages). Continuous variables were compared using Wilcoxon or paired Student *t*-tests and Mann–Whitney U-test or Student *t*-tests. Categorical variables were compared using Chi-2 or Fisher-exact tests.

We used four different methods to assess the association between SaO_2_ and SpO_2_: correlations, agreement, multivariable regression models and concordance. Specifically, correlations were performed using Pearson or Spearman’s correlation coefficients, according to data distribution. Agreement between SpO_2_ and SaO_2_ measurements was assessed with Bland–Altman analysis^33^ and intraclass correlation coefficients (ICC), with ICC value > 0.7 and > 0.9 indicating satisfactory and excellent agreement respectively^34^. Accuracy of SpO_2_ measurement was estimated by the bias, calculated as the mean difference between SpO_2_ and SaO_2_ measurements. Precision of SpO_2_ measurement was estimated by the standard deviation of the bias and the 95% limits of agreement^33^. The concordance between relative changes in SpO_2_ and SaO_2_ was assessed (i) with a four-quadrant plot analysis^35^, (ii) the clinical concordance method^36^ and (iii) the inter-rater agreement kappa coefficient (κ), with κ-value < 0.20 and > 0.80 indicating poor and good strength of agreement respectively^37^.

Multiple linear regressions were performed to identify variables associated with the dependent variable SpO_2_. Separate models were built depending on the selected variable (SaO_2_ or PaO_2_) in regard to SpO_2_. Moreover, multivariable models accounted for confounding variables (in regard to SpO_2_), and their interactions: SaO_2_ or PaO_2_, COVID-19 status, ethnic group, PEEP level, FiO_2_ setting, temperature, SpO_2_ probe and arterial catheter side (ipsilateral or contralateral), ARDS severity (as defined above, with mild category used as reference) and norepinephrine administration (defined as a categorical binary variable)^14–21^.

Assuming a correlation coefficient of 0.69 between SpO_2_ and SaO_2_^38^ and an ICC > 0.9 with a 95% confidence interval (CI) (0.84–0.96), we planned to include at least 50 patients with at least 25 COVID-19 patients. A* p* value < 0.05 was considered statistically significant. Statistical analyses were performed using MedCalc 11.6.0 software (MedCalc®, Mariakerke, Belgium), and SPSS version 25.0 (IBM®, Armonk, USA).

### Ethics approval and consent to participate

This study was approved by the Comité de Protection des Personnes Ile-de-France X (IDCRB2018-A00050-55, protocol 59–2018) and by the Ethics Committee of the Société de Réanimation de Langue Française (CE SRLF 20–72)^25^, which waived the need for informed consent, following national regulation on standard-of-care data collection. Indeed, this study was performed on data collected in the course of a prospective data collection, without any additional blood sample, as compared to standard of care. As such, only refusal to participate was systematically sought.

## Results

### Patient characteristics

Among 55 included patients: 33(60%) were men, 17(31%) were black and no patient had sickle-cell anaemia history. The ICU mortality rate was 38%. All patients had pulmonary ARDS and 26(47%) had an ARDS related to COVID-19. COVID-19 patients were more frequently black, tended to have more diabetes mellitus and had a lower SAPS-3 than non-COVID-19 patients (Table 1). The other characteristics of patients are shown in Tables 1 and 2.

**Table 1:** Patient characteristics.

Variables	All patients (n = 55)	COVID-19 patients (n = 26)	Non COVID-19 patients (n = 29)
Age (years)	60 (17)	59 (15)	60 (18)
Male (n, %)	33 (60)	17 (65)	16 (55)
Body mass index (kg/m^2^)	28 (5)	29 (5)	26 (5)
Black patients (n, %)	17 (31)	12 (46)	5 (17)*
Hypertension (n,%)	23 (42)	11 (42)	12 (41)
Diabetes mellitus (n,%)	14 (25)	10 (38)	4 (14)
Smokers (n,%)	18 (33)	7 (27)	11 (38)
SAPS-3	56 (15)	52 (11)	60 (17)*
ICU mortality (n, %)	21 (38)	7 (27)	14 (48)
Patients in sinus rhythm (n, %)	54 (98)	26 (100)	28 (97)
Patients receiving norepinephrine (n, %)	40 (73)	20 (77)	20 (69)
Neuromuscular blocking agents (n, %)	30 (55)	12 (46)	18 (62)
**SpO**_**2**_** probe location**			
Finger (n,%)	52 (95)	26 (100)	26 (90)
Ear (n,%)	3 (5)	0 (0)	3 (10)
**Arterial catheter location**			
Radial (n,%)	39 (71)	24 (92)	15 (52)*
Femoral (n,%)	16 (29)	2 (8)	14 (48)*

Variables are expressed as mean (standard deviation) or counts (percentages).

*ICU* Intensive Care Unit, *SAPS* Simplified Acute Physiology Score, *SpO*_*2*_ pulse oximetry.

*p < 0.05 non-COVID-19 versus COVID-19 patients.

**Table 2:** Respiratory, haemodynamic and oxygenation effects of the PEEP trial according to patients’ COVID-19 status.

Variables	COVID-19 patients (n = 26)		Non COVID-19 patients (n = 29)	
	PEEP + 5	PEEP + 15	PEEP + 5	PEEP + 15
**Respiratory parameters**				
Tidal volume (mL/kg of PBW)	5.9 [5.7–6.2]	5.9 [5.7–6.2]	5.9 [5.5–6.0]	5.9 [5.5–6.0]
Respiratory rate (cycles/min)	30 [26–33]	30 [26–33]	30 [25–30]	30 [25–30]
Total PEEP (cmH_2_O)	6 [5–6]	16 [15–16]*	5 [5–6]£	15 [15–16]*
Plateau pressure (cmH_2_O)	15 [14–17]	27 [24–28]*	18 [15–21]£	28 [26–29]*
Driving pressure (cmH_2_O)	9 [8–11]	10 [8–12]	13 [10–15]£	12 [10–14]£
Respiratory system compliance (mL/cmH_2_O)	44 [32–50]	38 [31–43]	28 [23–39]£	30 [28–37]
**Haemodynamic parameters**				
Heart rate (bpm)	80 [70–90]	78 [71–91]	93 [80–121]£	83 [75–117]£
Systolic arterial pressure (mmHg)	113 [102–131]	111 [103–120]	103 [92–122]	100 [93–127]
Diastolic arterial pressure (mmHg)	57 [50–61]	58 [50–62]	53 [51–57]	52 [49–57]£
Mean arterial pressure (mmHg)	75 [67–81]	74 [69–81]	71 [68–76]	69 [65–73]
Dosage of norepinephrine (µg/kg/min)	0.17 [0.10–0.28]	0.19 [0.12–0.39]£	0.50 [0.18–0.97]	0.50 [0.18–0.99]£
Lactate (mmol/L)	1.1 [0.8–1.2]	1.0 [0.8–1.2]£	1.8 [1.1–2.7]	1.7 [1.1–2.9]£
**Biological parameters**				
SpO_2_ (%)	95 [93–96]	96 [95–98]	91 [89–94]£	96 [93–98]*
SaO_2_ (%)	92 [89–95]	96 [94–98]*	90 [88–94]	96 [92–99]*
PaO_2_/FiO_2_ ratio	154 [99–225]	200 [152–250]*	101 [81–147]£	175 [110–211]*
PaO_2_ (mmHg)	69 [59–85]	87 [68–118]*	62 [58–73]	82 [65–126]*
PaCO_2_ (mmHg)	44 [41–50]	47 [42–51]	46 [41–50]	48 [41–52]*
Carboxyhaemoglobin (%)	0.8 [0.6–0.9]	0.7 [0.5–0.9]*	1.0 [0.8–1.5]£	0.9 [0.8–1.4]*£
Methaemoglobin (%)	0.7 [0.6–0.8]	0.7 [0.6–0.8]	0.6 [0.5–0.8]	0.6 [0.5–0.8]
Haemoglobin (g/dL)	11.8 [9.8–13.2]	11.9 [9.6–13.4]	9.7 [8.8–11.8]£	9.8 [8.5–11.6]£
Temperature (°C)	37.3 [36.8–38.0]	37.0 [37.0–37.8]	36.8 [36.4–37.5]£	36.8 [36.3–37.5]£
pH	7.38 [7.32–7.41]	7.36 [7.30–7.39]*	7.33 [7.30–7.38]	7.32 [7.29–7.35]*
Bicarbonates (mmol/L)	25.7 [22.8–28.0]	26.3 [22.4–28.4]	24.0 [21.7–27.1]	23.6 [21.7–27.2]
Fibrinogen (g/L)	6.8 [5.2–8.0]	NA	4.9 [3.6–6.5]^{1}^£	NA
D-Dimer (ng/mL)	1524 [977–2389]^{9}^	NA	4872 [NA]^{28}^	NA

Variables are expressed as median [interquartile]. No missing values, except otherwise specified between {}. NA: non-available.

*PaO*_*2*_ partial arterial pressure of oxygen, *PaCO*_*2*_ partial arterial pressure of carbon dioxide; PBW: predicted body weight, *PEEP* positive end-expiratory pressure, *SaO*_*2*_ arterial oxygen saturation, *SpO*_*2*_ pulse oximetry.

*p < 0.05 PEEP + 15 *vs.* PEEP + 5 cmH2O. £ *p* < 0.05 non-COVID-19 versus COVID-19 patients.

Formulas: driving pressure = plateau pressure-total PEEP; respiratory system compliance = tidal volume/(plateau pressure-total PEEP).

A SpO_2_ finger probe was used in all patients but three and a radial arterial catheter was used in 39(71%) patients. SpO_2_ probe and arterial catheter were located on the same side in 30(55%) patients (Table 1). SpO_2_ values ranged from 83 to 100% and SaO_2_ values from 80 to 100%. Carboxyhaemoglobin and methaemoglobin rates were < 2% in all patients.

### Association between SpO_2_, SaO_2_ and PaO_2_

**Figure 1:**
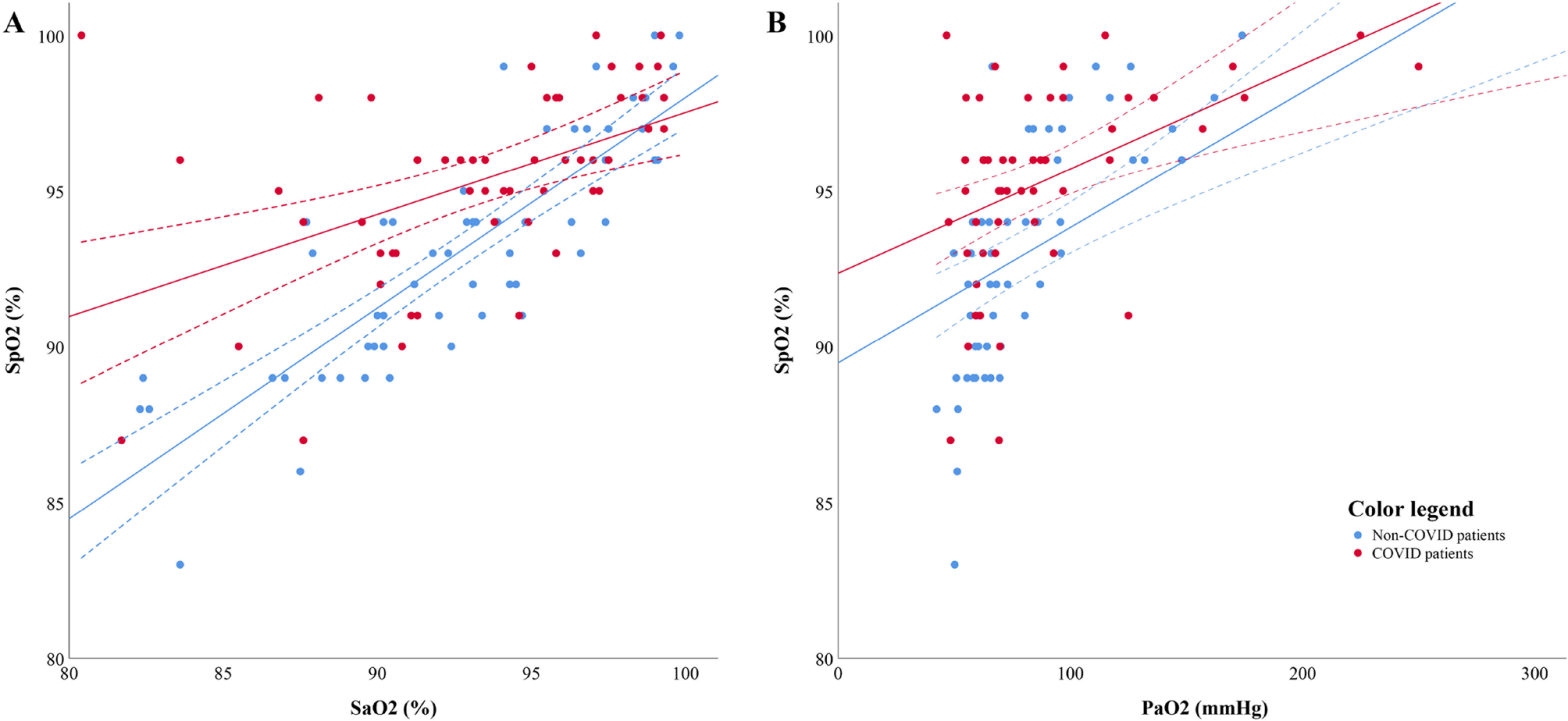
*Panel A*: correlation between pulse oximetry (SpO_2_) and arterial oxygen saturation (SaO_2_) in COVID-19 patients (red points, n = 52 measurements) and non-COVID-19 patients (blue points, n = 58 measurements). The solid line represents the correlation line. The dotted lines represent the 95% confidence interval of each correlation. *Panel B*: correlation between pulse oximetry (SpO_2_) and arterial oxygen partial pressure (PaO_2_) in COVID-19 patients (red points, n = 52 measurements) and non-COVID-19 patients (blue points, n = 58 measurements). The dotted lines represent the 95% confidence interval of each correlation.

In the whole population, SpO_2_ and SaO_2_ measurements were significantly correlated (r = 0.70, *p* < 0.0001). Correlation between SpO_2_ and SaO_2_ was lower in COVID-19 than in non-COVID-19 patients (r = 0.55, *p* < 0.0001 *vs.* r = 0.84, *p* < 0.0001, *p* = 0.002 for intergroup comparison) (Fig. 1A). After adjusting for confounding covariables, variables independently associated with SpO_2_ were: SaO_2_ (unstandardized β = 0.46, 95%CI = 0.35–0.57, *p* < 0.0001), COVID-19 status (unstandardized β = 1.96, 95%CI = 1.03–2.88 l, *p* < 0.0001) and PEEP level (unstandardized β = 0.11, 95%CI = 0.01–0.21, *p* = 0.03).

For sensitivity, correlation between SpO_2_ and PaO_2_ was also assessed and found significant (r = 0.62, *p* < 0.0001). Similarly, correlation between SpO_2_ and PaO_2_ was lower in COVID-19 than in non-COVID-19 patients (r = 0.47, *p*= 0.001 *vs.* r = 0.75, *p* <  0.0001, *p* = 0.002 for intergroup comparison) (Fig. 1B). After adjusting for confounding covariables, variables independently associated with SpO_2_ were: PaO_2_ (unstandardized β = 0.03, 95%CI = 0.02–0.05, *p* < 0.0001), COVID-19 status (unstandardized β = 2.04, 95%CI = 0.99–3.08, *p* < 0.0001) and PEEP level (unstandardized β = 0.17, 95%CI = 0.06–0.28, *p* = 0.002).

### Agreement between SpO_2_ and SaO_2_

**Figure 2:**
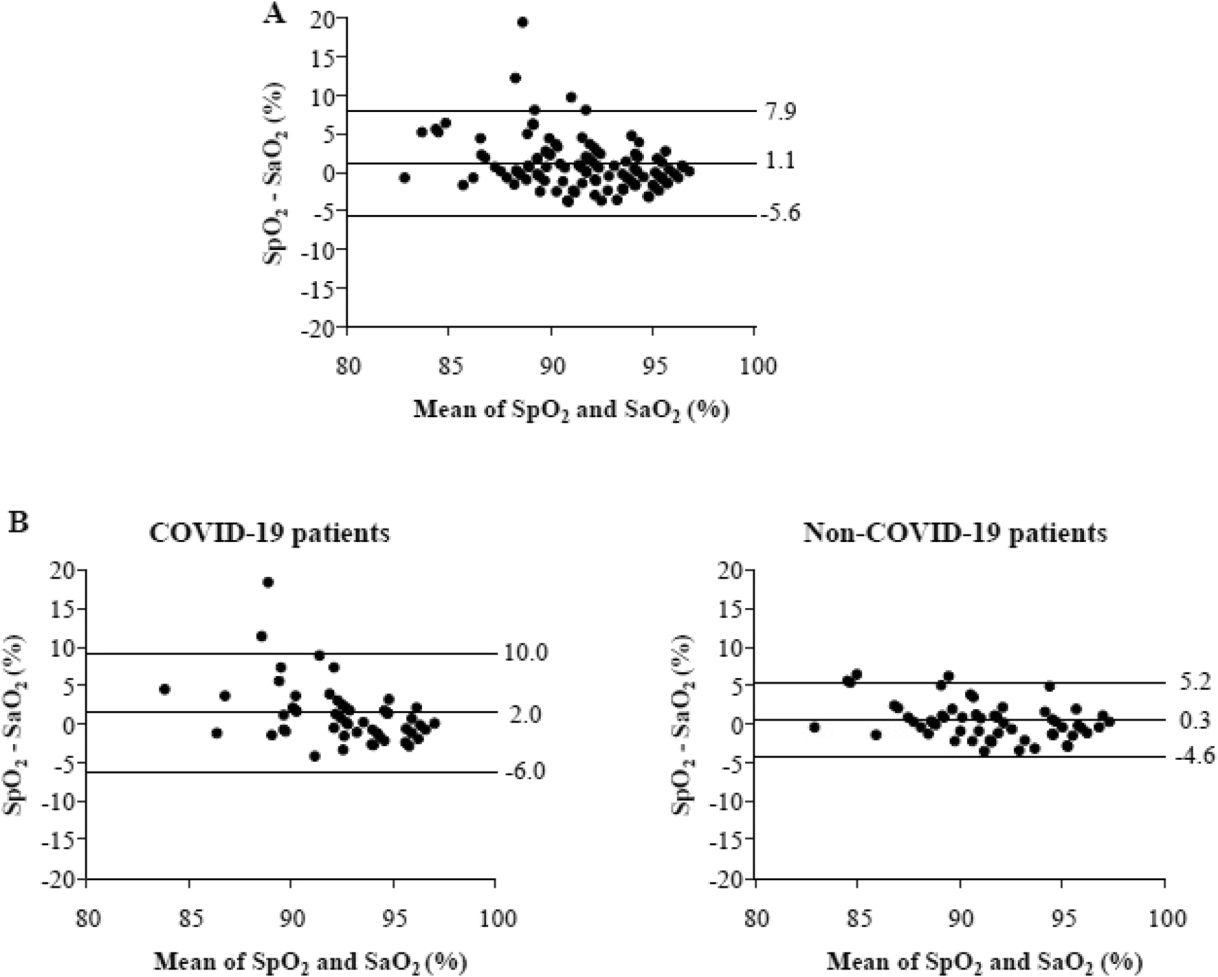
Comparison of pulse oximetry (SpO_2_) and arterial oxygen saturation (SaO_2_) measurements using the Bland–Altman method. The solid line represents the bias and the dotted lines represent the 95% limits of agreement (mean ± 1.96 standard deviation). *Panel A*: in the whole population (n = 110 measurements). *Panel B*: in COVID-19 patients (n = 52 measurements) and non-COVID-19 patients (n = 58 measurements).

In the whole population, the bias between SpO_2_ and SaO_2_ measurements was 1.1%, the precision was 3.4% and the limits of agreement ranged from -5.6 to + 7.9% (Fig. 2A). Bias and SaO_2_ measurements were significantly correlated (r = -− 0.56, *p *< 0.0001). The bias was higher (2.0 *vs.* 0.3%;* p* = 0.02) and the precision lower (4.1 *vs.* 2.5%) in COVID-19 than in non-COVID-19 patients (Fig. 2B). ICC = 0.79 (95%CI = 0.69–0.86), 0.62 (95%CI = 0.34–0.78) and 0.90 (95%CI = 0.84–0.94) in the whole population, in COVID-19 and in non-COVID-19 patients respectively. After adjusting for confounding covariables, COVID-19 status (unstandardized β = 1.77, 95%CI = 0.38–3.15, *p* = 0.01), ethnic group (unstandardized β = − 1.58, 95%CI = − 2.99 to − 0.18, *p* = 0.03) and ARDS severity (unstandardized β = 1.09, 95%CI = 0.21–1.97, *p* = 0.02) were independently associated with the bias. Interaction analyses did not yield significant association between COVID-19 status, ethnic group and bias.

Occult hypoxemia, occurred in 6 (5.5%) pairs of measurements. There was no significant difference between COVID-19 and non-COVID-19 patients (7.7% vs. 3.4%, respectively, *p* = 0.42).

### Effects of PEEP trial on SpO_2_, SaO_2_ and PaO_2_

**Figure 3:**
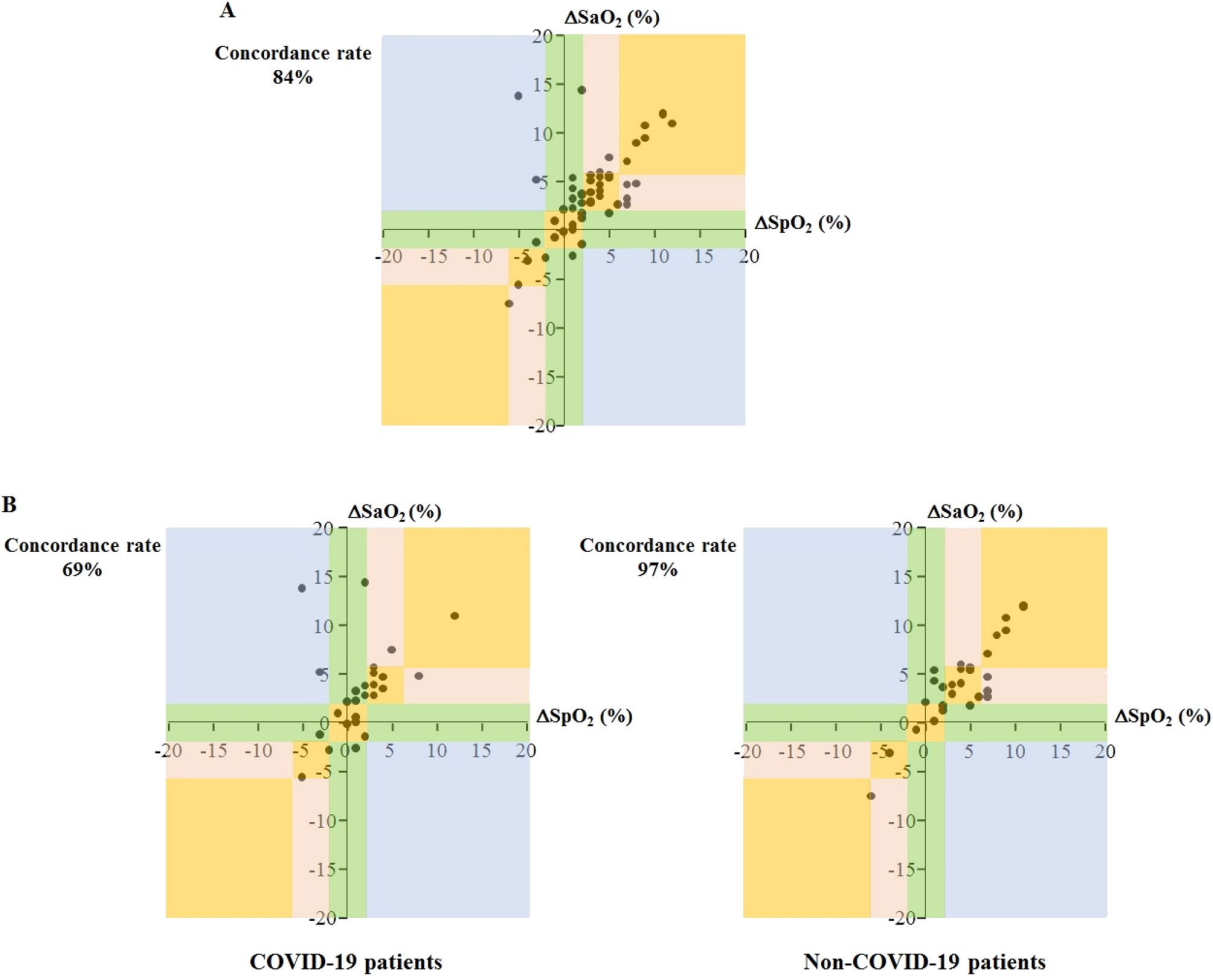
Trending ability of pulse oximetry (SpO_2_) against arterial oxygen saturation (SaO_2_) measurements during a positive end-expiratory pressure trial based on four-quadrant concordance analysis. The error grid reflects the therapeutic consequences in specific zones in the concordance plot. *Orange zone*: ΔSpO_2_ and ΔSaO_2_ change in the same direction and to the same extent. *Pink zone*: ΔSpO_2_ and ΔSaO_2_ change in the same direction but not to the same extent. *Green zone*: ΔSpO_2_ changes while ΔSaO_2_ is constant or vice versa. *Blue zone*: ΔSpO_2_ and ΔSaO_2_ change in opposite directions. *Panel A*: in the whole population (n = 55). *Panel B*: in COVID-19 (n = 26) and non-COVID-19 (n = 29) patients.

In the whole population, PEEP increase from 5 to 15 cmH2O significantly increased SpO_2_, SaO_2_ and PaO_2_ by 3 ± 4%, 4 ± 5% and 43 ± 57% respectively, (Table S1). SpO_2_ changes were significantly correlated to that of SaO_2_ (r = 0.65, *p* < 0.0001) and PaO_2_ (r = 0.47, *p* < 0.001). The concordance rate between PEEP trial-induced changes in SpO_2_ and SaO_2_ was 84% (Fig. 3A) and the κ-coefficient was 0.60. The concordance rate between PEEP trial-induced changes in SpO_2_ and PaO_2_ was 85% and the κ-coefficient was 0.55 (Figure S1).

Concordance rate between PEEP trial-induced changes in SpO_2_ and SaO_2_ was lower in COVID-19 than in non-COVID-19 patients (69 *vs.* 97%), as was the κ-coefficient (0.35 *vs.* 1.00) (Fig. 3B). Similar results were found for PaO_2_ (73 *vs.* 97% for concordance rate and 0.28 *vs.* 1.00 for the κ-coefficient) (Figure S1).

## Discussion

A reliable SpO_2_ measurement would be interesting in patients with ARDS, given the medico-economic impact of the decrease in arterial blood gas analysis overuse^10^. In this cohort of patients with ARDS monitored with last generation pulse oximeter, we found that (i) SpO_2_ was overall well-correlated with arterial oxygenation (SaO_2_ and PaO_2_) and that SpO_2_ slightly overestimated SaO_2_, (ii) COVID-19 status significantly impacted the association between SpO_2_ and arterial oxygenation (SaO_2_ and PaO_2_) and (iii), changes in SpO_2_ could not reliably track changes in arterial oxygenation (SaO_2_ and PaO_2_) during PEEP trial in COVID-19 patients.

The accuracy of SpO_2_ measurement in ICU is still debated because of several confounding factors^14–20,39,40^ with an unconstant and unpredictable bias between SpO_2_ and SaO_2_ measurements^14,15,18–20,39,41^. Overall, we found that SpO_2_ and arterial oxygenation (SaO_2_ and PaO_2_) were well-correlated and that SpO_2_ only slightly overestimated SaO_2_ but with wide limits of agreement, suggesting the lack of precision of SpO_2_ measurement. Our results are comparable to those from previous ICU studies, which found similar correlation^38^ and a bias ranging from 0.2 to 3%^14,15,38,40,42^ with a mean bias of 1.5%^43^ but wide limits of agreement^14,15,38,40,42^, highlighting the lack of improvement in the last generation pulse oximeter reliability. We also confirmed that that SpO_2_ measurement was still less reliable in black^21^ and in most hypoxemic^14–18^ patients. It has been very recently shown that black patients had nearly three times the frequency of occult hypoxemia that was not detected by SpO_2_ measurement as Caucasian patients^21^. Conversely to previous studies^14,39^, we also found that changes in SpO_2_ could track changes in arterial oxygenation (SaO_2_ and PaO_2_) during PEEP trial with a moderate to good strength of agreement. To our knowledge, this is one of the first studies analyzing not only the magnitude (correlation) but also the direction (concordance rate and κ-coefficient) of SpO_2_ and SaO_2_ changes. The discrepancy of our results to those of previous studies may be explained by the fact that rather than considering multiple therapeutic manoeuvres^15,40^, we only considered a PEEP trial to ensure that the possible lack of agreement between changes in SpO_2_ and SaO_2_ could not be attributed to the multiple therapeutic manoeuvres used.

We found that COVID-19 significantly impacted the association between SpO_2_ and arterial oxygenation (SaO_2_ and PaO_2_). First, SpO_2_ measurement was less reliable, as illustrated be weaker correlation, higher bias, wider limits of agreement, lower precision and lower ICC with an ICC < 0.7. Second, changes in SpO_2_ could not reliably track changes in arterial oxygenation (SaO_2_ and PaO_2_) during PEEP trial, with a concordance rate of 69% and a κ-coefficient of 0.35 for changes in SaO_2_ and a concordance rate of 73% and a κ-coefficient of 0.28 for changes in PaO_2_ in COVID-19 patients, but a concordance rate of 97% and a κ-coefficient of 1.00 both for SaO_2_ and PaO_2_ in non-COVID-19 patients. To our knowledge, this is the first study investigating the reliability of SpO_2_ measurement in COVID-19 patients, despite it has been suggested to use SpO_2_ monitoring in these patients to detect “silent hypoxemia”^44,45^. Importantly, the impact of COVID-19 on the association between SpO_2_ and arterial oxygenation cannot be explained solely by the fact that COVID-19 patients were predominantly black^22^. Moreover, the proportion of occult hypoxemia that we observed was that expected of our population, although no thorough analysis could be performed on this criterion due to its low prevalence. Indeed, although lack of power may have been involved, interaction analyses did not yield significant association between COVID-19 status, ethnic group and SpO_2_. With caution, this may suggest that COVID-19 per se may alter the accuracy of SpO_2_ measurement and the agreement between SpO_2_ and arterial oxygenation. This could be explained by the fact that COVID-19 patients may experience systemic microvascular alterations that appear to be common and linked to coagulopathy^24^ and/or endothelial dysfunction and endotheliitis^46^. Indeed, it has been shown that SARS-CoV-2 infection may facilitate the induction of endotheliitis with viral elements within endothelial cells and accumulation of inflammatory cells leading to apoptosis found in in several organs^46^. Further studies are needed to address the potential impact of COVID-19 on SpO_2_ measurement.

The clinical implications of our results are two-fold. In non COVID-19 patients, our results suggest that SpO_2_ measurements with latest generation pulse oximeter may be sufficient to monitor and track changes in arterial oxygenation in the most critically-ill patients. Thus, arterial blood gazes may not be necessarily mandatory after every ventilatory setting modification during the weaning process in patients with ARDS. Moreover, our results confirm those published by Martínez-Balzano who showed that there was an overuse of arterial blood gazes in critically ill patients and that their number could be reduced without negatively impacting patient care but with clear medico-economic impact^10^. Meanwhile, in COVID-19 patients, SpO_2_ measurement may not be as reliable, and iterative arterial blood gazes’ analyses to measure SaO_2_ may still be mandatory.

Our study has some limitations. First, we assessed only one type of last generation pulse oximeter. Second, we only considered the same two PEEP levels in all patients and we cannot exclude that considering other PEEP levels might influence the association between SpO_2_ and SaO_2_ as well as the concordance between PEEP trial-induced SpO_2_ and SaO_2_ changes. Third, we did not study, as previous studies^18,40,41^, the influence of carboxyhaemoglobin and methaemoglobin on the accuracy of SpO_2_ measurement. Indeed, pulse oximeters cannot differentiate between these two forms of haemoglobin and oxyhaemoglobin, leading to SaO_2_ overestimation in patients with high carboxyhaemoglobin and/or methaemoglobin rates^47,48^. Yet, we systematically excluded patients with methaemoglobinemia or carbon monoxide poisoning (all had carboxyhaemoglobin and methaemoglobin rates < 2%). Interestingly, Kerget and colleagues very recently showed that endogenous carboxyhaemoglobin may be an easily accessible biomarker of clinical course and prognosis in COVID-19 patients^49^. Fourth, we did not perform multiple arterial blood gas analyses, which may have allowed the estimation of the metrological precision feature of the pulse oximeter device. Indeed, random error variability of replicate measurements, defined as precision, usually requires to perform multiple measurements for a given timepoint^50^. In our study, we did not deem ethical to sample arterial blood gases several times for each datapoint. We pragmatically assumed that precision remained constant and random error measurements were reproducible, following the device specifications^51^.

## Conclusion

In patients with ARDS, SpO_2_ was associated with arterial oxygenation (SaO_2_ and PaO_2_) and could track changes in arterial oxygenation with good reliability. Nevertheless, COVID-19 status significantly impacted the association between SpO_2_ and arterial oxygenation.

## Supplementary Information

Supplementary Information.

## Data Availability

The datasets used and/or analyzed during the current study are available from the corresponding author on reasonable request.
