# Supplementary material for: Impact of COVID-19 on the association between pulse oximetry and arterial oxygenation in patients with acute respiratory distress syndrome
Source: Sci Rep. 2022 Jan 27;12:1462. doi: 10.1038/s41598-021-02634-z (PMC8795352; doi:10.1038/s41598-021-02634-z)
Supplement: Supplementary file 1 — Supplementary Information. [file 41598_2021_2634_MOESM1_ESM.docx]

***Table S1. Respiratory, haemodynamic and oxygenation effects of PEEP trial in the whole population.***

| **Variables** | **PEEP +5** | **PEEP +15** |
| --- | --- | --- |
| **Respiratory parameters** |  |  |
| Tidal volume (mL/kg of PBW) | 5.9 [5.7-6.1] | 5.9 [5.7-6.1] |
| Respiratory rate (cycles/min) | 30 [25-32] | 30 [25-32] |
| Total PEEP (cmH2O) | 6 [5-6] | 16 [15-16]* |
| Plateau pressure (cmH2O) | 16 [15-19] | 27 [25-29]* |
| Driving pressure (cmH2O) | 11 [8-14] | 11 [10-13] |
| Respiratory system compliance (mL/cmH2O) | 33 [25-50] | 33 [27-41] |
| **Haemodynamic parameters** |  |  |
| Heart rate (bpm) | 87 [74-103] | 86 [73-104] |
| Systolic arterial pressure (mmHg) | 106 [99-124] | 104 [95-123]* |
| Diastolic arterial pressure (mmHg) | 56 [50-58] | 55 [49-60] |
| Mean arterial pressure (mmHg) | 73 [62-80] | 72 [66-79] |
| Dosage of norepinephrine (µg/kg/min) | 0.27 [0.13-0.66] | 0.37 [0.15-0.66]* |
| Lactate (mmol/L) | 1.2 [0.9-2.0] | 1.2 [0.9-2.1] |
| **Biological parameters** |  |  |
| SpO_2_ (%) | 93 [90-95] | 96 [93-98]* |
| SaO_2_ (%) | 91 [88-95] | 96 [92-98]* |
| PaO_2_/FiO_2_ ratio | 115 [84-174] | 177 [119-215]* |
| PaO_2_ (mmHg) | 66 [58-80] | 84 [65-123]* |
| PaCO_2_ (mmHg) | 44 [41-50] | 47 [41-52]* |
| Carboxyhaemoglobin (%) | 0.9 [0.7-1.2] | 0.8 [0.7-1.2]* |
| Methaemoglobin (%) | 0.7 [0.6-0.8] | 0.7 [0.6-0.8] |
| Haemoglobin (g/dL) | 10.9 [9.0-12.0] | 11.0 [9.0-13.0] |
| Temperature (°C) | 37.0 [36.5-37.9] | 37.0 [36.8-37.6] |
| pH | 7.36 [7.31-7.39] | 7.33 [7.29-7.38]* |
| Bicarbonates (mmol/L) | 24.6 [22.6-27.6] | 25.2 [21.9-27.8] |

n=55, variables are expressed as median [interquartile].

* p<0.05 PEEP +15 *vs.* PEEP +5 cmH2O.

Formulas: driving pressure=plateau pressure-total PEEP; respiratory system compliance= tidal volume/(plateau pressure-total PEEP)

Abbreviations: PaO_2_: partial arterial pressure of oxygen; PaCO_2_: partial arterial pressure of carbon dioxide; PBW: predicted body weight; PEEP: positive end-expiratory pressure; SaO_2_: arterial oxygen saturation; SpO_2_: pulse oximetry


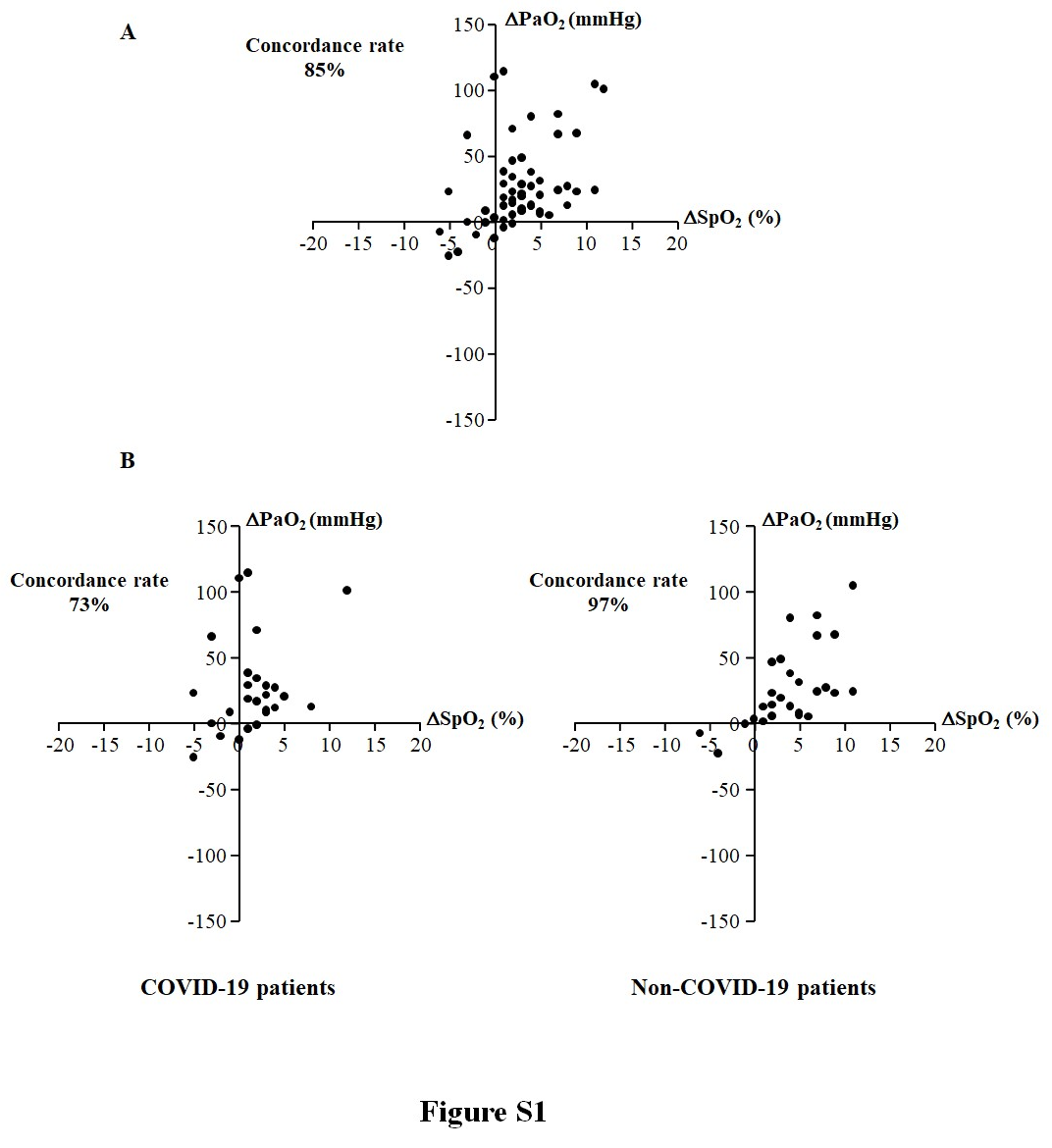


**Supplemental Figure Legends**

**Figure S1**

Trending ability of pulse oximetry (SpO_2_) against arterial oxygen partial pressure (PaO_2_) measurements during a positive end-expiratory pressure trial based on four-quadrant concordance analysis. *Panel A*: in the whole population (n=55). *Panel B*: in COVID-19 (n=26) and non-COVID-19 (n=29) patients.
